# Graves’ disease following immunosuppression withdrawal and SARS-CoV-2 infection in a failed kidney allograft recipient: a case report on immune reconstitution

**DOI:** 10.3389/fimmu.2026.1826575

**Published:** 2026-08-11

**Authors:** Nan Yang, Xian Wu, Li Zhao, Shouci Hu, Cong Xia, Zhiyu Li, Qingqing Ye

**Affiliations:** 1 Department of Nephrology, The First Affiliated Hospital of Zhejiang Chinese Medical University (Zhejiang Provincial Hospital of Chinese Medicine), Hangzhou, Zhejiang, China; 2 Zhejiang Key Laboratory of Research and Translation for Kidney Deficiency-Stasis-Turbidity Disease, Zhejiang-Macau International Joint Laboratory of Integrated Traditional Chinese and Western Medicine for Nephrology and Immunology, Hangzhou, Zhejiang, China; 3 The Second Clinical Medical College, Zhejiang Chinese Medical University, Hangzhou, Zhejiang, China; 4 Zhejiang Provincial People’s Hospital, Affiliated to Hangzhou Medical College, Hangzhou, Zhejiang, China

**Keywords:** B-cell lymphopenia, Graves’ disease, immune reconstitution inflammatory syndrome, immunosuppression withdrawal, SARS-CoV-2 infection, Thyrotropin receptor antibodies

## Abstract

Immune recovery after withdrawal of long-term immunosuppression may unmask autoimmune disease, but thyroid autoimmunity in patients with failed kidney allografts remains poorly characterized. We report a 29-year-old man with kidney allograft failure who developed severe acute respiratory syndrome coronavirus 2 (SARS-CoV-2) infection after discontinuation of maintenance immunosuppression and subsequently presented with weight loss, tremor, tachycardia, diffuse goiter, and overt thyrotoxicosis. Positive thyrotropin receptor antibodies (TRAb) and diffusely increased radionuclide uptake supported a diagnosis of Graves’ disease rather than destructive thyroiditis. His symptoms and thyroid function improved following treatment with methimazole, propranolol, glucocorticoids, and supportive treatment. Extracorporeal blood-purification therapies were used in the setting of persistent tachycardia, dialysis-related blood pressure fluctuations, and systemic inflammation, although their independent contribution could not be determined. Persistently elevated TRAb levels despite low peripheral B-cell counts suggested that circulating B-cell numbers did not reliably reflect humoral autoimmune activity. This case highlights a possible immune reconstitution–associated form of thyroid autoimmunity after immunosuppression withdrawal and emphasizes early recognition and thyroid evaluation following new inflammatory triggers.

## Introduction

Kidney transplantation requires long-term immunosuppressive therapy to maintain allograft survival, with broad effects on T- and B-cell activation and immune regulation (1). After allograft failure, the optimal timing and sequence of immunosuppression tapering remain uncertain, and the downstream immunologic consequences are incompletely characterized (2, 3). Chronic allograft rejection typically evolves gradually and is a major cause of kidney allograft failure (4). Immune recovery after graft failure and immunosuppression withdrawal may not simply restore the pre-transplant baseline and may be accompanied by persistent immune perturbations (2, 3, 5).

Graves’ disease (GD) is an autoimmune thyroid disorder characterized by hyperthyroidism, and its development is closely associated with the persistent production of autoantibodies against the thyroid-stimulating hormone (TSH) receptor, commonly termed thyrotropin receptor antibodies (TRAb). The generation of TRAb is generally considered to result from the interplay of multiple factors, including genetic susceptibility, infectious triggers, and alterations in immune status. These factors may contribute to autoreactive immune responses through disruption of immune tolerance mechanisms.

Autoimmune disorders have also been reported during states of immune reactivation, such as immune checkpoint inhibitor (ICI) therapy (6). By blocking immune checkpoint molecules and thereby breaking peripheral tolerance, these therapies frequently induce immune-related adverse events (irAEs). ICI- associated thyroid dysfunction occurs in approximately 2%-15% of patients and has been associated with T-/B-cell dysregulation and autoantibody production (7, 8). However, GD after kidney allograft failure with immunosuppression withdrawal has not been systematically described. Autoimmune thyroid disease has been described after discontinuation of maintenance immunosuppression in islet transplant recipients previously exposed to lymphocyte-depleting induction. In a 2019 case series, five recipients developed autoimmune thyroid disease, including four cases of GD, 11–18 months after withdrawal of maintenance immunosuppression (9).This finding suggests that cessation of immunosuppressive therapy may represent a potential trigger for GD. Viral infections have been implicated in the initiation or amplification of thyroid autoimmunity (10). How immunosuppression withdrawal and viral infection may interact to sustain TRAb production and drive GD remains unclear. In this case, peripheral B-cell counts were low despite persistent autoantibody production, prompting consideration of possible mechanisms.

We report a patient who developed GD after withdrawal of long-term immunosuppression and subsequent SARS-CoV-2 infection, with persistent TRAb elevation despite peripheral B-cell lymphopenia. This report aims to describe the clinical course and diagnostic findings, consider an immune reconstitution-like interpretation without asserting causality, and emphasize practical strategies for early recognition.

## Case description

The patient was a 29-year-old man with end-stage kidney disease attributed to hypertension receiving maintenance hemodialysis since 2017. In February 2018, he underwent kidney transplantation and was subsequently maintained on long-term immunosuppressive therapy with mycophenolate mofetil (MMF), tacrolimus, and prednisone, with stable allograft function for several years. He had no prior history of thyroid disease, and thyroid function had remained normal throughout routine follow-up. He had no known family history of thyroid disease, autoimmune disorders, or other relevant hereditary conditions. He denied smoking, alcohol abuse, and other relevant toxic exposures. Since 2023, allograft function gradually declined, accompanied by recurrent respiratory infections. In April 2024, he underwent endovascular repair for Stanford type B aortic dissection. Thereafter, allograft function further declined; immunosuppression was tapered and withdrawn, and maintenance hemodialysis was resumed. Before the onset of thyrotoxicosis, immunosuppressive therapy had already been withdrawn. Low-dose tacrolimus and prednisone were reintroduced during hospitalization.

In August 2025, the patient developed fever, cough, nasal congestion, rhinorrhea, and myalgia. SARS-CoV-2 infection was confirmed by a positive rapid antigen test. Chest computed tomography showed no evidence of pneumonia. Shortly thereafter, he developed progressive weight loss, insomnia, fatigue, psychomotor agitation, increased bowel frequency, and poorly controlled hypertension during hemodialysis. Because these symptoms persisted and progressed, he was admitted for further evaluation. On admission, the patient appeared anxious and agitated. His vital signs were as follows: temperature 37.3 °C, blood pressure 150/94 mmHg, heart rate 109 beats/min, respiratory rate 17 breaths/min, and oxygen saturation 98%. He was 176 cm tall and weighed 63.6 kg. Physical examination revealed diffuse thyroid enlargement without palpable nodules, a fine tremor of the outstretched hands, warm and moist skin, and brisk deep tendon reflexes. No obvious thyroid tenderness or definite signs of Graves’ ophthalmopathy were noted. During hemodialysis, tachycardia persisted and was accompanied by labile hypertension. Tests showed overt hyperthyroidism, with elevated thyroid hormones and suppressed TSH (**Table 1**). The TRAb level was elevated. Thyroid ultrasonography showed diffuse enlargement with coarse, heterogeneous echotexture and no obvious focal lesion. Thyroid scintigraphy showed diffuse enlargement with markedly increased technetium uptake, without definite cold or hot nodules. Overall findings were most consistent with GD. Inflammatory markers were also elevated, consistent with systemic inflammation. Destructive thyroiditis related to viral infection was also considered. However, positive TRAb, diffuse goiter, and markedly increased technetium uptake supported a diagnosis of GD rather than destructive thyroiditis.

**Table 1:** Dynamic changes in thyroid function, inflammatory markers, and coagulation parameters during hospitalization and follow-up.

Laboratory parameter	At admission	Pre-CRRT	Post-CRRT	At discharge	Follow-up	Reference range	Unit
FT3	>30.72	25.26	4.79	4.10	3.72	2.43–6.01	pmol/L
FT4	>64.35	>64.35	20.73	17.20	12.69	9.01–19.05	pmol/L
TT3	>9.22	4.28	1.16	1.20	1.17	0.98–2.33	nmol/L
TT4	>308.88	>308.88	131.15	135.92	95.69	62.68–150.84	nmol/L
TSH	<0.01	0.01	0.01	0.01	0.87	0.35–4.94	mIU/L
TRAb	12.70	14.30	9.92	7.34	5.42	0–2	IU/L
IL-6	13.92	—	—	7.13	5.35	0–6.28	pg/mL
TNF-α	8.08	—	—	5.58	3.15	0–5.12	pg/mL
ESR	29	—	—	17	8	0–15	mm/h
D-dimer	7.99	5.29	5.47	4.75	1.21	0–0.55	mg/L FEU

FT3, free triiodothyronine; FT4, free thyroxine; TT3, total triiodothyronine; TT4, total thyroxine; TSH, thyroid-stimulating hormone; TRAb, thyrotropin receptor antibodies; IL-6, interleukin-6; TNF-α, tumor necrosis factor-α; ESR, erythrocyte sedimentation rate; CRRT, continuous renal replacement therapy. Pre-CRRT and Post-CRRT indicate laboratory assessments immediately before and after CRRT initiation, respectively. Follow-up refers to the first outpatient reassessment after discharge. “—” indicates that the parameter was not assessed.

After the diagnosis was confirmed, antithyroid and supportive therapy were initiated. Methimazole 15 mg once daily and propranolol 20 mg three times daily were administered for thyrotoxicosis and adrenergic symptom control. Hydrocortisone (50 mg every 12 h) was administered from September 1 to September 3, 2025 because of concern for severe thyrotoxicosis and possible impending thyroid storm. Low-dose prednisone acetate (7.5 mg once daily) and tacrolimus (0.5 mg once daily) were reintroduced during hospitalization as maintenance immunosuppression in the context of prior renal allograft failure. Additional supportive treatment, including antihypertensive and phosphate-lowering therapy, was provided. Meanwhile, traditional Chinese medicine was used as adjunctive therapy under physician supervision, and the patient did not use non-prescribed herbal medications. Given persistent tachycardia, labile blood pressure, poor tolerance of hemodialysis and systemic inflammation, intensified blood purification was initiated, including hemoperfusion, intermittent hemodialysis, and continuous renal replacement therapy (CRRT). The details of the blood purification modalities, duration, and timing are summarized in **Table 2**.

**Table 2:** In-hospital timeline of blood purification therapies.

Date	Treatment	Modality	Duration	Purpose
2025-08-26	Admission	—	—	Initial assessment of disease severity
2025-08-27	Hemodialysis	HD	4 h	Metabolic and fluid control
2025-08-28	Hemoperfusion	HP	2 h	Adjunctive removal of inflammatory mediators
2025-08-29	Hemodialysis	HD	4 h	Fluid management
2025-08-30	Continuous renal replacement therapy	CRRT (CVVHDF)	10 h	Intensified supportive therapy; enhanced inflammatory control; maintenance of hemodynamic and metabolic stability
2025-08-31	Continuous renal replacement therapy	CRRT (CVVHDF)	10 h	Consolidation of therapeutic effects
2025-09-01	Hemodialysis	HD	4 h	Maintenance therapy
2025-09-03	Hemodialysis	HD	4 h	Maintenance therapy

HD, hemodialysis; HP, hemoperfusion; CRRT, continuous renal replacement therapy; CVVHDF, continuous veno-venous hemodiafiltration. Duration refers to the actual duration of each blood purification intervention. “—” indicates not applicable.

The patient tolerated methimazole, propranolol, hydrocortisone, and blood purification well during hospitalization, without documented treatment interruption or major treatment-related adverse events. No major adverse drug reactions or unexpected complications related to antithyroid therapy, β-blockade, hydrocortisone, or blood purification were observed during hospitalization. Serial monitoring showed a progressive decline in thyroid hormone levels, with inflammatory markers returning to their respective reference ranges (**Table 1**). Clinically, tachycardia, gastrointestinal symptoms, and dialysis-related blood pressure fluctuations improved. During follow-up, thyroid function continued to improve, TRAb levels gradually decreased, and thyroid hormone levels ultimately returned to the normal range.

## Discussion

**Figure 1:**
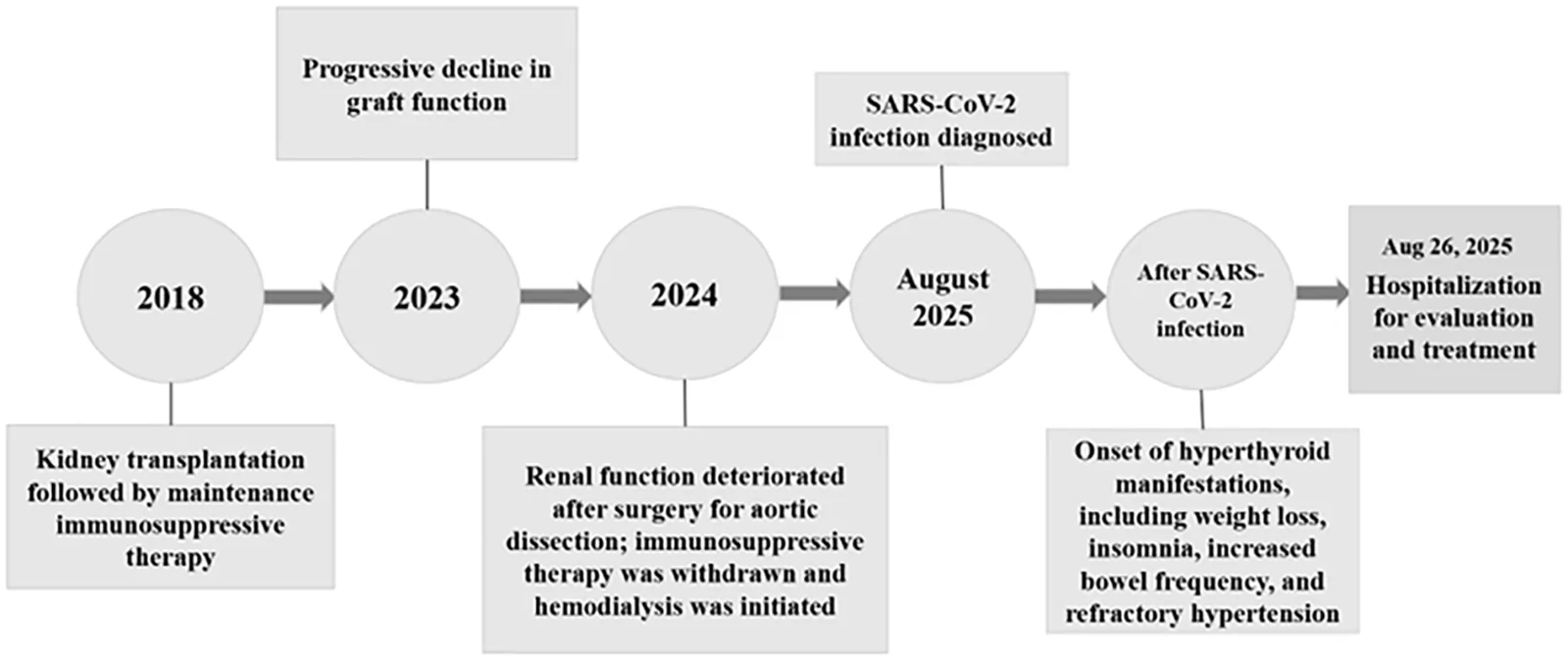
Clinical timeline of disease progression and key events in the present case. The timeline summarizes the major clinical events, including kidney transplantation, progressive graft failure, withdrawal of immunosuppressive therapy, SARS-CoV-2 infection, onset of hyperthyroid manifestations, and subsequent hospitalization for evaluation and treatment.

Supported by the clinical timeline (**Figure 1**) and serial laboratory findings (**Table 1**), this case describes the development of GD after withdrawal of long-term immunosuppression in a patient with kidney allograft failure and no known prior thyroid disease. An additional notable feature was the persistence of markedly elevated TRAb levels despite low peripheral B-cell counts. The sequence of immunosuppression withdrawal, subsequent SARS-CoV-2 infection, and GD onset demonstrates a temporal association but does not establish causality. Autoimmune thyroid disease has previously been reported after discontinuation of maintenance immunosuppression in islet transplant recipients exposed to lymphocyte-depleting induction and during immune reconstitution following alemtuzumab therapy, suggesting that immune recovery may provide a permissive context for thyroid autoimmunity (9, 11). However, evidence regarding this phenomenon in patients with failed kidney allografts remains scarce. Together with evidence linking viral infection to thyroid autoimmunity (10), the present case raises the possibility that immune recovery after immunosuppression withdrawal and SARS-CoV-2-related inflammation may have acted as complementary contributors to the development of GD.

Dynamic laboratory monitoring (**Table 1**) showed severe thyrotoxicosis at admission, accompanied by markedly elevated TRAb and systemic inflammatory markers. After initiation of antithyroid therapy and intensified blood purification (including hemoperfusion and CRRT), both thyroid function indices and inflammatory markers improved in parallel. During follow-up, thyroid function normalized and TRAb levels progressively declined. The parallel improvement in thyroid indices and inflammatory markers coincided with clinical recovery but does not establish that systemic inflammation amplified the autoimmune response. Although CRRT and hemoperfusion were employed with the intent of clearing inflammatory mediators, their specific contribution to the observed improvement beyond supportive management remains speculative and cannot be definitively established from this single case.

This case may be interpreted within an immune reconstitution inflammatory syndrome (IRIS)-like framework. Autoimmune thyroid disease during immune reconstitution has been most extensively described after antiretroviral therapy in people with human immunodeficiency virus (HIV) and following lymphocyte-depleting therapy with alemtuzumab, with GD being a recognized and often delayed manifestation (11–13). The present case similarly involved autoimmune disease emerging during presumed immune recovery; however, immune reconstitution followed withdrawal of long-term immunosuppression rather than CD4^+^ T-cell recovery after antiretroviral therapy. Restoration of T-cell help to autoreactive B cells is biologically plausible, although the underlying mechanism remains uncertain. SARS-CoV-2-associated inflammation may have provided an additional trigger. These observations raise the possibility of an IRIS-like phenomenon may occur after immunosuppression withdrawal in patients with failed kidney allografts.

In this case, withdrawal of maintenance immunosuppression- including tacrolimus, MMF, and prednisone- may have contributed to immune reconstitution- associated dysregulation by affecting different lymphocyte subsets. Tacrolimus, a calcineurin inhibitor, suppresses interleukin-2- dependent T-cell activation; its withdrawal may lead to recovery of T-cell activation, restoring T-cell help to autoreactive B cells. MMF inhibits lymphocyte proliferation via blockade of *de novo* purine synthesis, and its discontinuation may permit recovery of lymphocyte populations. Prednisone exerts broad inhibitory effects on innate and adaptive immunity, and its withdrawal may further enhance global immune activation (1). Together, these changes may contribute to autoreactive B-cell responses and autoantibody production, potentially facilitating overt GD in a post-transplant IRIS-like context.

Another notable immunologic feature of this case was the discordance between markedly reduced peripheral B-cell counts and persistently elevated TRAb levels. Peripheral B-cell enumeration reflects the circulating compartment and may not fully represent ongoing antibody production in bone marrow or thyroid tissue. One possible explanation is continued TRAb secretion by long-lived plasma cells residing in specialized survival niches, as these cells can maintain antibody production independently of circulating B-cell pools. Selected antibody responses have also been shown to persist despite sustained depletion of circulating CD19^+^ B cells (14, 15). In addition, recent single-cell analyses of GD thyroid tissue have identified local T-B-cell interactions and atypical or pathogenic B-cell populations, indicating that tissue immune activity may not be adequately captured by peripheral blood measurements (16). Withdrawal of immunosuppression might also have altered T-cell help and the functional composition of the B-cell compartment. In GD, reduced interleukin-10 (IL-10)-producing regulatory B cells and expansion of CD11c^+^ B cells capable of differentiating into antibody-secreting cells have been reported (17, 18).These mechanisms provide biologically plausible explanations for the observed dissociation; however, long-lived plasma cells, intrathyroidal antibody production, and restored T-cell help were not directly evaluated in this patient. Therefore, the persistent elevation of TRAb despite low circulating B-cell counts suggests that peripheral B-cell numbers may not reliably reflect humoral autoimmune activity, but it does not establish any specific cellular mechanism.

The temporal link with SARS-CoV-2 infection suggests that the virus may have acted as an inflammatory trigger, pushing an already vulnerable immune state into overt autoimmunity. SARS-CoV-2 infection has been associated with immune dysregulation and autoantibody production and may act as an inflammatory trigger for new-onset or unmasked autoimmune disease in susceptible individuals. Proposed mechanisms include immune hyper-stimulation, molecular mimicry between host and viral antigens, neutrophil extracellular traps, and virus-driven transcriptional changes in immune genes (19). The elevated interleukin-6 (IL-6) and erythrocyte sedimentation rate (ESR) at admission were consistent with systemic inflammation but were nonspecific. However, without comprehensive cytokine profiling or antigen-specific functional assays, the exact molecular link between COVID-19 and GD onset remains speculative and needs further study.

In light of the clinical course, this case suggests that a failed kidney allograft itself may represent a distinct immunological state. Long-term immunosuppression may have attenuated the clinical expression of an underlying autoimmune susceptibility, although this possibility cannot be confirmed because baseline thyroid autoantibody data were unavailable. However, during immune reconstitution following withdrawal of immunosuppressive therapy, recovery in immune cell numbers may not be fully synchronized with restoration of humoral immune regulation, potentially leading to emergence or amplification of an antibody-mediated autoimmune response. This interpretation is consistent with the temporal sequence of immunosuppression withdrawal, SARS-CoV-2 infection, and GD onset and suggests a possible role for post-transplant immune remodeling.

Several limitations warrant consideration. First, as a single-case report, this study cannot establish causality; the temporal association between immunosuppression withdrawal, SARS-CoV-2 infection, and GD onset does not demonstrate a definitive mechanistic link. Second, although we propose that the discordance between low peripheral B-cell counts and elevated TRAb levels may reflect contributions from non-circulating antibody-secreting cells, such as long-lived plasma cells in survival niches, this remains speculative in the absence of direct cellular or molecular evidence. The lack of high-parameter flow cytometry, single-cell multi-omics, and functional B- and T-cell assays limits definitive characterization of the lymphocyte subsets involved. Future prospective studies incorporating serial immunophenotyping, cytokine profiling, and, when feasible, single-cell RNA sequencing coupled with variable (V), diversity (D), and joining (J) gene repertoire analysis are needed. Third, direct evaluation of long-lived plasma cells and tissue-resident lymphocytes would have required bone marrow or thyroid sampling, which was not clinically indicated. Fourth, although CRRT and hemoperfusion were used as adjunctive extracorporeal therapies, evidence for such modalities in severe thyrotoxicosis is largely limited to case reports and case series, and their specific contribution beyond standard antithyroid and supportive management remains uncertain (20). In addition, the detailed diagnostic work-up, close serial monitoring, and extracorporeal support available at our center may not be readily reproducible in hospitals with more limited resources, which may restrict the generalizability of the management approach. Therefore, this case should not be interpreted as supporting the routine use of hemoperfusion or CRRT; rather, its broadly applicable clinical value lies in early recognition, basic thyroid evaluation, and timely referral when severe thyrotoxicosis is suspected. Finally, the patient received traditional Chinese medicine as adjunctive therapy. Because the pharmacologic effects of this regimen were not systematically evaluated, its potential effects on thyroid function or interactions with concomitant medications cannot be excluded.

## Conclusion

This case suggests that GD may develop following withdrawal of long-term immunosuppression in the setting of kidney allograft failure. A plausible explanation is that immune reconstitution after immunosuppression withdrawal may be temporally and functionally heterogeneous, potentially allowing dysregulated humoral immunity and the emergence of autoantibody-mediated thyroid autoimmunity in a post-transplant immune reconstitution-like state. This interpretation is temporally consistent with the patient’s stable thyroid function during prolonged immunosuppression, followed by markedly elevated TRAb levels and overt thyrotoxicosis after withdrawal. Although a causal relationship cannot be established, the temporal association with SARS-CoV-2 infection raises the possibility that viral immune activation contributed to or exacerbated underlying immune dysregulation. Clinical improvement was observed during multimodal treatment, including antithyroid therapy and supportive blood purification, although the specific contribution of extracorporeal therapies remains uncertain.

This case underscores the potential clinical relevance of immune reconstitution-related autoimmunity in patients with failed kidney allografts after discontinuation of immunosuppressive therapy. In symptomatic patients with failed allografts after immunosuppression withdrawal-especially after an inflammatory trigger-prompt thyroid-function testing may facilitate earlier recognition of evolving thyrotoxicosis.

## Patient perspective

The patient reported marked physical discomfort and psychological distress after immunosuppression withdrawal and subsequent coronavirus disease 2019 (COVID-19) infection. He experienced persistent weight loss, insomnia, and severe fatigue, which interfered with his ability to tolerate regular hemodialysis and caused significant anxiety. Following antithyroid treatment and intensified blood purification, his symptoms gradually improved, with recovery of sleep, bowel function, and more stable blood pressure during dialysis. At follow-up, he reported a full return to normal daily activities. The patient provided written informed consent for publication of his clinical course, hoping it may offer insight for others with failed kidney allografts and immune-related complications.

## Data Availability

The original contributions presented in the study are included in the article/supplementary material. Further inquiries can be directed to the corresponding authors.
